# Surface proteins involved in the adhesion of *Streptococcus salivarius* to human intestinal epithelial cells

**DOI:** 10.1007/s00253-018-8794-y

**Published:** 2018-02-13

**Authors:** Fanny Chaffanel, Florence Charron-Bourgoin, Claire Soligot, Mounira Kebouchi, Stéphane Bertin, Sophie Payot, Yves Le Roux, Nathalie Leblond-Bourget

**Affiliations:** 1 DynAMic, Université de Lorraine, INRA, 54506 Vandoeuvre-lès-Nancy, France; 2 UR AFPA, équipe PB2P, Université de Lorraine, INRA, 54506 Vandoeuvre-lès-Nancy, France

**Keywords:** *Streptococcus salivarius*, Adhesion, HT29-MX, Caco-2/TC7, Streptococcal surface proteins

## Abstract

The adhesion properties of 14 *Streptococcus salivarius* strains to mucus (HT29-MTX) and non-mucus secreting (Caco-2/TC7) human intestinal epithelial cells were investigated. Ability to adhere to these two eukaryotic cell lines greatly differs between strains. The presence of mucus played a major factor in adhesion, likely due to high adhesiveness to mucins present in the native human mucus layer covering the whole cell surface. Only one *S. salivarius* strain (F6-1), isolated from the feces of a healthy baby, was found to strongly adhere to HT-29 MTX cells at a level comparable to that of *Lactobacillus rhamnosus* GG, a probiotic strain considered to be highly adherent. By sequencing the genome of F6-1, we were able to identify 36 genes encoding putative surface proteins. Deletion mutants were constructed for six of them and their adhesion abilities on HT-29 MTX cells were checked. Our study confirmed that four of these genes encode adhesins involved in the adhesion of *S. salivarius* to host cells. Such adhesins were also identified in other *S. salivarius* strains.

**Electronic supplementary material:**

The online version of this article (10.1007/s00253-018-8794-y) contains supplementary material, which is available to authorized users.

## Introduction

*Streptococcus salivarius* is a Gram-positive bacterium that is closely related to *Streptococcus thermophilus* and *Streptococcus vestibularis* (Delorme et al. 2015; Pombert et al. 2009). Although some *S. salivarius* strains were reported to be opportunistic pathogens associated with sporadic cases of meningitidis (Wilson et al. 2012), endocarditis (Kitten et al. 2012), or bacteremia (Corredoira et al. 2005), the majority of *S. salivarius* strains act as commensal bacteria that colonize mucosal surfaces of humans (Aas et al. 2005).

*S. salivarius* is a natural inhabitant of the oropharyngeal and gastrointestinal tracts (GIT) of humans (Carlsson et al. 1970; Park et al. 2005; Wang et al. 2005). It colonizes the human oral cavity soon after birth and remains there as a predominant inhabitant. *S. salivarius* often constitutes the majority of total cultivable flora on soft tissues of the mouth and in saliva and is a major component of biofilms colonizing the dorsal surface of the tongue and the buccal epithelium (Bowden et al. 1979). *S. salivarius* is also naturally present in the upper part of the digestive tract (Qin et al. 2010), especially in the stomach and jejunum where it persists throughout the human life (Hakalehto et al. 2011; Van den Bogert et al. 2013; Wang et al. 2005). In human, these commensal bacteria play important ecological roles. Together with the other bacteria of the resident microbiota, they form a barrier against pathogens and reduce their adhesion and colonization. *S. salivarius* strains also display antimicrobial activities against virulent streptococci and therefore contribute to the maintenance of oral, pharyngeal, and gut health (Patras et al. 2015; Burton et al. 2013; Santagati et al. 2012; Wescombe et al. 2006; Caufield et al. 2000). They also display immunomodulation properties by inhibiting inflammatory pathways of human epithelial cells activated by the pathogens (Kaci et al. 2014; Tagg and Dierksen 2003). Therefore, adhesion of *S. salivarius* to oral and intestinal mucosa seems crucial for its interaction with the host and maintenance of homeostasis of the human oral and gastrointestinal microbiota.

The binding of bacteria to human cells involves specific adhesins that are mainly surface proteins (Desvaux et al. 2006). These are held at the cell surface either (i) covalently by sortases (e.g., LPXTG proteins) or (ii) non-covalently (e.g., proteins with a LysM motif or cell wall-binding domains). They may also be tethered to the cell membrane through N-terminal modifications with lipid (lipoproteins). Up to now, the attachment process of *S. salivarius* to host mucosal surface or to other bacteria has been insufficiently studied.

*S. salivarius* is one of the streptococcal species with the greatest number of extracellular proteins (Delorme et al. 2015), but only a few of them have been demonstrated to play a role in *S. salivarius* adhesion. These include a 27 kDa protein and the glycoprotein AgC that are responsible for binding to the salivary-glycoprotein EP-GP (Schenkels et al. 1993) and to buccal epithelial cells, respectively (Weerkamp and Jacobs 1982; Weerkamp and McBride 1980). Furthermore, CspB is associated with fimbriae (Lévesque et al. 2004), which are observed on up to 50% of the *S. salivarius* strains in the human oral cavity (Handley et al. 1999) and are involved in the coaggregation of *S. salivarius* with other bacteria (Lévesque et al. 2003). Fimbriae participate in the establishment of microbial communities by recognizing specific receptors in their natural environments (Kline et al. 2009; Fernández and Berenguer 2000). Another surface appendage, the fibrils, is important for *S. salivarius* adhesion to oral host cells and interbacterial coaggregation (Handley et al. 1984, 1987). Fibrils are also found in *Streptococcus gordonii*, *Streptococcus sanguinis*, and *Streptococcus oralis* (Elliott et al. 2003).

In this context, the first aim of the present study was to evaluate the auto-aggregation abilities of 14 strains of *S. salivarius*, a phenotype considered important for the adhesion of probiotic bacteria to intestinal epithelial cells (Lukić et al. 2012; Del Re et al. 2000). The second aim was to study the adhesion abilities of these strains to GIT cell lines and to clarify a possible link between auto-aggregation and adhesion properties of these bacterial strains. Two distinct human epithelial cell lines isolated from colonic carcinoma were used because they mimic GIT cells: (i) Caco-2/TC7 cells differentiate in vitro spontaneously in polarized cells forming a confluent cell monolayer mimicking the enterocytes of the small intestine and expressing cell surface proteins found in these cells. Furthermore, Caco-2/TC7 cells lack mucus secretion (Liévin-Le Moal and Servin 2013; Hilgendorf et al. 2000) and (ii) HT29-MTX cells differentiate in vitro into goblet cells and produce a lot of mucus mainly composed of MUC5AC mucin, predominantly produced in the stomach (Liévin-Le Moal and Servin 2013; Lesuffleur et al. 1990). Our last objective was to identify proteins playing a role in GIT colonization. For that purpose, knockout mutants of six genes encoding surface proteins of the most adherent strain F6-1 were generated and used to test their involvement in auto-aggregation and in adhesion to epithelial cells.

## Materials and methods

### Bacterial strains and growth conditions

In previous works, we examined the susceptibility of commensal and clinical *S. salivarius* strains to various antibiotics and we searched for the presence of integrative and conjugative elements in their genomes (Chaffanel et al. 2015; Dahmane et al. 2017). Here, 14 of these strains were further characterized: B35, F1-4, F1-8, F4-2, F6-1, L64, N20, T93 (Dahmane et al. 2017; Chaffanel et al. 2015), B50, B63, L10, L25, L61, and S6-13 (Chaffanel et al. 2015). They were grown at 37 °C without shaking in M17 medium (Terzaghi and Sandine 1975) supplemented with glucose (10 g L^−1^) (hereafter denoted as GM17) and conserved at − 20 °C in GM17 supplemented with 20% (*w*/*v*) of glycerol (Sigma-Aldrich, Saint Quentin Fallavier, France). Either erythromycin (5 μg mL^−1^) or spectinomycin (500 μg mL^−1^) (Sigma-Aldrich, Saint Quentin Fallavier, France) was added to GM17 medium for the selection of mutants.

*L. rhamnosus* GG (LGG) (ATCC® 53103^™^) was included as a positive control (Laparra and Sanz 2009) and was grown as previously described by Kebouchi et al. (2016). Bacterial cultures were grown for 13 h (stationary phase) before storage and use for auto-aggregation assay or adhesion experiments.

*Escherichia coli* strains (Table 1) used for plasmid propagation were routinely grown in Luria-Bertani (LB) medium (Sambrook and Russell 2006) at 37 °C with shaking, supplemented with erythromycin (100 μg mL^−1^) or spectinomycin (50 μg mL^−1^). The F6-1 strain characterized further in this study has been deposited at the CIRM (Centre International de Ressources Microbiennes, Nouzilly, France) under the accession number CIRMBP-1193.

**Table 1:** The *S. salivarius* F6-1 deletion mutants created in this study and bacterial strains and plasmids used for their construction

Strains and plasmids	Genotype	Origin/reference
*Streptococcus salivarius* mutants		
F6-1 ∆srtA	Complete deletion of the *sortase A* gene, Em^R^	This study
F6-1 ∆0442	1530-bp internal deletion of the F6-1 *SALIVA_0442* gene, Spec^R^	This study
F6-1 ∆0576	478-bp internal deletion of the F6-1 *SALIVA_0576* gene, Spec^R^	This study
F6-1 ∆1457	> 4856-bp internal deletion of the F6-1 *SALIVA_1457* gene, Spec^R^	This study
F6-1 ∆1472	218-bp internal deletion of the F6-1 *SALIVA_1472* gene, Spec^R^	This study
F6-1 ∆1473	5854-bp internal deletion of the F6-1 *SALIVA_1473* gene, Spec^R^	This study
F6-1 ∆1475	> 3683-bp internal deletion of the F6-1 *SALIVA_1475* gene, Spec^R^	This study
*Escherichia coli*		
DH5α	*supE44 lacU169* (ϕ80 *lacZ* M15) *hsdR17 endA1 gyrA96 thi-1relA1*	Taylor et al. (1993)
EC101	*supE hsd-5 thi* (*lac-proAB*) F (*traD6 proAB lacI*^q^*lacZ* M15) *repA*, derivative of strain TG1	Leenhouts (1995)
Plasmids		
pG^+^host9	Thermosensitive plasmid with pVE6002 replication origin, Em^R^	Maguin et al. (1996)
pSL1180 spec lox	Plasmid pSL1180 with the *Spe*I-*Spe*I spectinomycin resistance cassette amplified from the pSET4S plasmid and cloned in its *Avr*II site, Amp^R^, Spec^R^	This study
pSET4s	Thermosensitive plasmid with pVE6002 and ColE1 replication origin, LacZ’, Spec^R^	Takamatsu et al. (2001)
pSL1180	Plasmid with pBR322 replication origin, Amp^R^	Amersham, Piscataway, NJ, USA

Amp^R^ ampicillin resistance, Em^R^ erythromycin resistance, Spec^R^ spectinomycin resistance

### Auto-aggregation assay

The cultures (5 mL) of the 14 *S. salivarius* strains were centrifuged at 4500 rpm for 10 min, and the pellets were washed three times with 5 mL of a peptone water solution (casein peptone 0.1% (*p*/*v*); Merck Millipore, Molsheim, France). The pellets were then resuspended in peptone water solution to obtain bacterial suspensions adjusted to OD_600nm_ = 0.8. A volume of 5 mL of these cultures was incubated at 37 °C for 5 h in a steady position. Aggregation was scored positive when clearly visible sand-like particles formed due to the precipitation of cells at the bottom of the tubes, leaving a clear supernatant. The aggregation ability was confirmed by turbidimetric methods at *λ* = 600 nm. The aggregation percentage was calculated by the following expression:

Auto-aggregation coefficient time at 5h (ACT) = 1 − (OD_t0_ of the bacterial suspension / OD_t5_ of the upper suspension after 5-h incubation) × 100.

Auto-aggregation assays were performed in triplicate. Negative controls (without bacteria) confirmed the medium sterility.

### DNA isolation and manipulation

Genomic DNA was isolated from *S. salivarius* F6-1 cells by a standard protocol for Gram-positive bacteria after lysozyme (10 mg mL^−1^)/mutanolysin (700 u mL^−1^) treatment of bacterial cells (Colmin et al. 1991). Plasmid DNA extraction from *E. coli* and standard DNA manipulations were performed according to established procedures (Sambrook and Russell 2006). Restriction enzymes were purchased from Fermentas (Waltham, MA, USA). PCR reagents and enzymes were purchased from Thermo Scientific (Villebon sur Yvette, France). Oligonucleotides (Supplementary Table S1) were obtained from Eurogentec (Seraing, Belgium). PCR and overlapping PCR products were purified prior to subsequent manipulation using Gene JET Gel Extraction Kit from Thermo Scientific (Villebon sur Yvette, France) or dialyzed on a 0.025-μm Millipore filter (Merck Millipore, Molsheim, France) using 5 mL of distilled water for 10 min at room temperature, respectively.

### Construction of *S. salivarius* mutants

Seven *S. salivarius* F6-1 mutants (Table 1) were constructed by allelic replacement (Supplementary Fig. S1). Briefly, the erythromycin and spectinomycin resistance genes were amplified from pG+host9 and pSL1180 spec lox plasmids, respectively (Table 1). In parallel, two DNA fragments (named 1 and 2 on Supplementary Fig. S1) of about 1000 bp overlapping the upstream and downstream regions of the gene to be deleted were amplified by PCR using specific primers that present an extended sequence matching with the 3′ and 5′ ends of the chosen resistance gene (Supplementary Table S1). A second PCR amplification was carried out using these two PCR fragments and the resistance gene as template to synthesize an overlap PCR product carrying the antibiotic resistance gene flanked by the upstream and downstream chromosomal regions of the gene to be deleted. This final PCR product was used to transform the F6-1 strain after addition of the synthetic peptide (H2N–LPYFTGCL–COOH, Proteogenix, Schiltigheim, France) allowing natural transformation (Fontaine et al. 2010). The crossover events, upstream and downstream from the gene to be deleted, were positively selected by the newly acquired antibiotic resistance of the transformed clones. Deletion mutants were confirmed by PCR and sequencing. PCRs were performed with 50 ng of genome or plasmid DNA or PCR products, 200 μM of each deoxynucleotide triphosphate (dNTP), 1 μM of each primer (for primer sequences, see Supplementary Table S1), and 0.02 U μL^−1^ of Phusion high-fidelity DNA polymerase (Thermo Scientific, Villebon sur Yvette, France) in appropriate buffer per 50 μL reaction volume. Classic PCR amplifications were performed using the following cycling parameters: 5 min at 98 °C, followed by 30 cycles of 30 s at 98 °C, 30 s at 58 °C and 1 min at 72 °C, and finally 5 min at 72 °C. Cycling conditions for the overlap PCR were 3 min at 98 °C, 15 cycles of three steps (10 s at 98 °C, incrementing for 30 s from 45 °C to 60 °C with 1°C at each cycle and 1 min 30 s at 72 °C), 30 cycles of three steps (10 s at 98 °C, 30 s at 58 °C, and 1 min 30 s at 72 °C), and a final extension for 10 min at 72 °C. The quantity and the size of amplified products were verified by electrophoresis on 1% agarose gels.

### Epithelial cell lines and culture conditions

Two different intestinal epithelial cell lines were used in this study: (i) the non-mucus-secreting Caco-2/TC7 cells (Liévin-Le Moal and Servin 2013; Hilgendorf et al. 2000) which were obtained from Pr. Isabelle Chevalot (UMR 7274, Laboratoire Réactions et Génie des Procédés, Nancy) and (ii) the mucus-secreting HT29-MTX cells (Liévin-Le Moal and Servin 2013; Lesuffleur et al. 1990) which were kindly provided by Dr. Thécla Lesuffleur (INSERM UMR S 938, Paris, France) (Lesuffleur et al. 1990). They were grown and conserved as previously described by Kebouchi et al. (2016). Seeding and transepithelial electrical resistance measurements were done as described by Kebouchi et al. (2016).

### Adhesion assay

Adhesion experiments were performed as previously described by Kebouchi et al. (2016) with minor modifications: the pellets of bacteria were washed once with 10 mL of peptone water (casein peptone 0.1% (*p*/*v*), Merck) and re-suspended in Dulbecco’s Modified Eagle Medium without antibiotics at a final concentration of 10^9^ CFU mL^−**1**^ (OD_600nm_ of about 12) for *S. salivarius* strains and 10^10^ CFU mL^−**1**^ (OD_600nm_ of about 90) for LGG. The initial number of viable bacterial cells was determined by plating on GM17 agar for *S. salivarius* and MRS agar (de Man Rogosa and Sharpe agar; Sigma-Aldrich, Saint Quentin Fallavier, France) for LGG.

### Statistical analysis

Experimental data were analyzed using a one-way analysis of variance (ANOVA) followed by a Tukey’s test. R 3.1.2 software was used for the statistical analysis. Results were expressed as means ± SEM. Differences were considered statistically significant when *P* value <0.05. For the bacterial adhesion of F6-1 mutants, the comparison was performed with the wild-type F6-1 strain.

### DNA sequencing and sequence analysis

Genomes of 12 *S. salivarius* strains used in this work were sequenced using an Illumina HiSeq2000 sequencer by Beckman Coulter Genomics (2 × 100 bp after paired-end library construction, at least 60× coverage). De novo assemblies were performed using CLC Genomics Workbench (CLC Bio) using default parameters. Scaffold of the genomes was built by using the Genome Finishing module of CLC Genomics Workbench with the *S. salivarius* JIM8777 genome as reference. Some assembly gaps were filled by doing PCR and sequencing. Multifasta files (one per genome) representing all translated CDSs of the studied genomes were generated. Among proteins, the ones being putatively secreted proteins were identified by their peptide signal sequence using the software Signal P 4.1 Server (http://www.cbs.dtu.dk/services/SignalP). Amino acid domains of each putative surface protein were retrieved with the CD-search tool from NCBI (www.ncbi.nlm.nih.gov/Structure/bwrpsb/bwrpsb.cgi) (Marchler-Bauer and Bryant 2004) to identify those known to be involved in bacterial adhesion. For phylogenetic analysis, the proteins were aligned using Clustal omega with default parameters (Sievers et al. 2011). The trees were built with MEGA (Tamura et al. 2013) using the ML method based on the JTT matrix-based model (Jones et al. 1992). The branch support of the groupings was estimated using bootstrap (100 replicates).

### Nucleotide sequence accession numbers

The sequences of the complete putative surface proteins have been deposited in the GenBank database under accession numbers MF497539 to MF497549 for F6-1 SALIVA_1475 homologs, MF497550 to MF497560 for F6-1 SALIVA_1473 homologs, MF497561 to MF497571 for F6-1 SALIVA_1472 homologs, and MF497572 to MF497580 for F6-1 SALIVA_1457 homologs. The F6-1 genome has been deposited at DDBJ/ENA/GenBank under the accession NPDJ00000000.

## Results

### Auto-aggregation and adhesion of *S. salivarius* strains

**Fig. 1:**
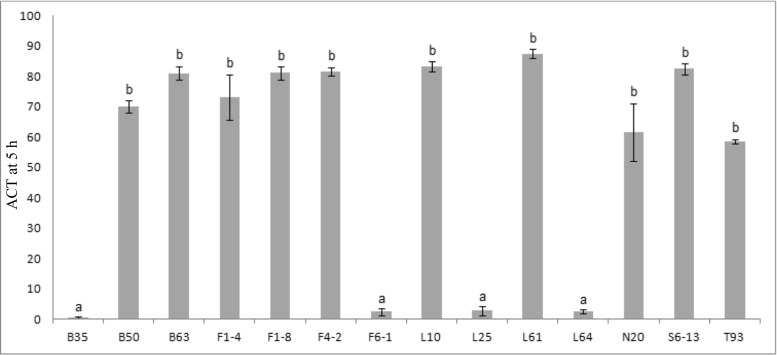
Auto-aggregation coefficient time (ACT) at 5 h for 14 *S. salivarius* strains. Data represent means ± SEM from experiments performed for each strain in triplicate. Lowercase letters (a, b) indicate the two significantly different groups of strains (*P* < 0.05)

The auto-aggregation abilities of 14 *S. salivarius* strains (Fig. 1) show two statistically different groups of strains (*P* < 0.05). One group includes the four *S. salivarius* strains B35, F6-1, L25, and L64, which show an auto-aggregation coefficient time (ACT) at 5 h below 5%. The other group contains 10 strains (B50, B63, F1-4, F1-8, F4-2, L10, L61, N20, S6-13, and T93) characterized by an ACT at 5 h higher than 58%.

**Fig. 2:**
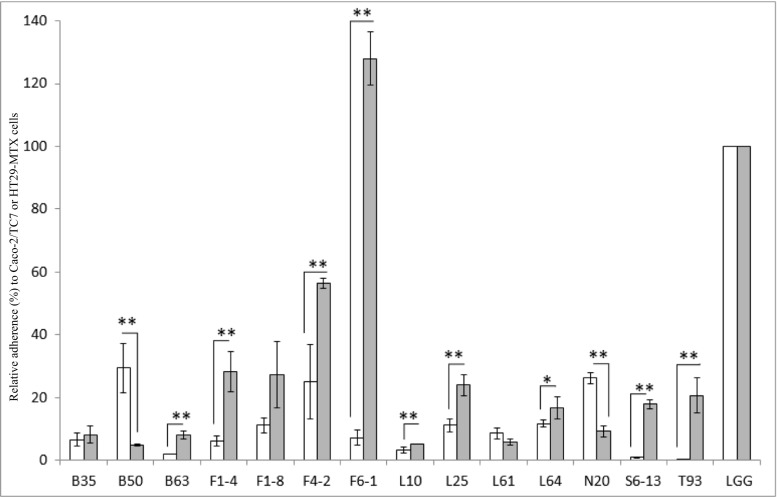
Adhesion capacity of 14 *S. salivarius* strains to Caco-2/TC7 (in white) and HT29-MTX (in gray) cell lines. *L. rhamnosus* GG (LGG) was used as a reference strain. The results are presented as a relative adherence calculated from the number of *S. salivarius* adhering bacteria (CFU per Caco-2/TC7 cell) compared to LGG which is standardized to 100%. Data represent means ± SEM from experiments performed for each strain in triplicate. * indicates 0.05 < *P* < 0.1 and ** indicates *P* < 0.05 for comparison of adhesion capacities on Caco-2/TC7 and HT29-MTX cells for each strain

The adhesion abilities of these 14 *S. salivarius* strains were tested on two human intestinal epithelial cell lines, Caco-2/TC7 (non-mucus secreting) and HT29-MTX (secreting mucus). Results presented in Fig. 2 are expressed as relative adherence on each cell line compared to the reference strain *L. rhamnosus GG* (LGG) and show that the *S. salivarius* strains display statistically different adhesion capacities. The less adherent strains are T93 on Caco-2/TC7 (0.30 ± 0.04 CFU per Caco-2/TC7 cell) and B50 on HT29-MTX (4.9 ± 0.4 CFU per HT29-MTX cell). The most adherent strains are B50 on Caco-2/TC7 (29.4 ± 7.8 CFU per Caco-2/TC7 cell) and F6-1 on HT29-MTX (128.0 ± 8.6 CFU per HT29-MTX cell). All the *S. salivarius* strains show an adhesion capacity to Caco-2/TC7 and HT29-MTX cells lower than LGG (for example, B50 is 3.4 and 20.4 times less adherent than LGG on Caco-2/TC7 and HT29-MTX cells, respectively), except F6-1, which shows an adhesion capacity to HT29-MTX cells which is 1.3 times more than LGG (Fig. 2).

Figure 2 also shows, for each *S. salivarius* strain, a comparison of its adhesion capacities on both Caco-2/TC7 and HT29-MTX cells. According to the results, 10 strains (B50, B63, F1-4, F4-2, F6-1, L10, L25, N20, S6-13, and T93) present statistically different adhesion capacities with the two cell lines (*P* < 0.05) (Fig. 2). The L64 strain trends to have a significant difference of adhesion capacity to the two cell lines (0.05 < *P* < 0.1). Most of these 11 strains, except B50 and N20, showed better adhesion ability to HT29-MTX cells than to Caco-2/TC7 cells. The three other strains (B35, F1-8, and L61) display no significant difference of adhesion capacity to both cell lines.

### Identification of F6-1 putative adhesins

F6-1 is the *S. salivarius* strain with the best adhesion capacity to the HT29-MTX cells, presumably due to the surface adhesins. Thus, a focus was made on this commensal strain, which was isolated from the feces of a healthy baby of 15 months. To be present on the outside of the bacterial cell, adhesins have to translocate across the cytoplasmic membrane before being exposed at the cell surface. Genes encoding putative surface proteins with a signal peptide were first identified in the genome sequence of F6-1. Thirty-six CDS encoding such proteins have been detected in the genome of F6-1 (Table 2). Nine of them have a classic signal peptide, 12 others have an YSIRK motif in their signal peptide, and 15 have a KxKYxGKY motif in their signal peptide. Among these 36 CDS, 10 present an LPxTG motif which is recognized by the sortase enzyme known to be involved in the cell surface anchorage (Navarre and Schneewind 1999). The 26 other CDS do not present any recognizable anchorage motif.

**Table 2.** Characteristics of the 36 putative surface proteins of *S. salivarius* F6-1

CDS name	F6-1 genome locus tag	Domain 1			Domain 2			Domain 3		
		Name		Number	Name		Number	Name		Number
Anchorage with LPxTG motif (TIGR01167)										
F6-1 Big_3-4^a,d^	CGZ74_07470	**collagen_bind**	PF05737	1						
	CGZ74_07465									
F6-1 CDS_143^a^	CGZ74_01075	Surf_Exclu_PgrA	TIGR04320	1	RnfC_N	PF13375	1			
F6-1 CDS_1475^a^	CGZ74_08635									
F6-1 CDS_812^b^	CGZ74_04900	**GbpC**	PF08363	1	DUF4337	PF14235	1	ProTailRpt	TIGR04307	1
F6-1 CDS_813^b^	CGZ74_04905	**GbpC**	PF08363	1	SMC_N	PF02463	1	ProTailRpt	TIGR04307	1
F6-1 SALIVA_0893^b,d^	CGZ74_04505	**CshA_fibril_rpt**	TIGR04225	27	repeat_SSSPR51	TIGR04308	2			
	CGZ74_04510									
F6-1 SALIVA_1472^b^	CGZ74_07440	5_nucleotid_C	PF02872	1	**MucBP**	PF06458	1			
F6-1 SALIVA_1473^b^	CGZ74_07445	Peptidase_M26_C	PF07580	1	Peptidase_M26_N	PF05342	1	**G5**	PF07501	2
F6-1 SALIVA_1456^c,d^	CGZ74_07350	**ser_adhes_Nterm**	TIGR04224	1	hyperosmo_Ebh	TIGR04264	2			
F6-1 SALIVA_1457^c,d^	CGZ74_07355 CGZ74_07360	**ser_adhes_Nterm**TIGR04224	1							
Anchorage without LPxTG motif										
F6-1 CDS_131^a^	CGZ74_01015	5_nucleotid_C	PF02872	1						
F6-1 CDS_769^a^	CGZ74_04650	CotH	PF08757	1	FN3	PF00041	1			
F6-1 CDS_800^a^	CGZ74_04830	MurNAc-LAA	cd02696	1	FlgJ	COG1705	1	**GBS-Bsp-like**	PF08481	2
F6-1 CDS_889^a^	CGZ74_05340	AraC	COG2207	1	AraC_binding	PF02311	1			
F6-1 CDS_925^a^	CGZ74_05580	**SSURE**	PF11966	3	DNA_pol3_gamma3	PF12169	1			
F6-1 CDS_1235^a^	CGZ74_07270	glucan_65_rpt	TIGR04035	7	endomucin	PF07010	1			
F6-1 CDS_283^b^	CGZ74_01820	Glyco_hydro_68	PF02435	1	DUF4045	PF13254	1	RNase_E_G	PF10150	1
F6-1 CDS_628^b^	CGZ74_03840	CBM6-CBM35-CMB36_like	cd14490	1	Herpes_ICP4_C	PF03585	1	Beta_helix_1	TIGR03805	1
F6-1 CDS_859^b^	CGZ74_05145	PulA	cl25948	1	IsdB	cl27124	1			
F6-1 SALIVA_0442^b^	CGZ74_02350	**GBS-Bsp-like**	PF08481	2	**SH3_5**	PF08460	6			
F6-1 SALIVA_0518^b^	CGZ74_02365	VanY	PF02557	1	**GBS-Bsp-like**	PF08481	4	**SH3_5**	PF08460	9
F6-1 SALIVA_0576^b^	CGZ74_02955	**FctA**	PF12892	4	**collagen_bind**	PF05737	1			
	CGZ74_07480									
F6-1 SALIVA_1475^b,d^	CGZ74_07485	**Antigen_C**	PF16364	10	**FctA**	PF12892	17			
	CGZ74_07490									
F6-1 CDS_708^c^	CGZ74_04295	Glyco_hydro_70	PF02324	1	glucan_65_rpt	TIGR04035	7			
F6-1 CDS_709^c^	CGZ74_046300	Glyco_hydro_70	PF02324	1	glucan_65_rpt	TIGR04035	5			
F6-1 CDS_715^c^	CGZ74_04330	Glyco_hydro_70	PF02324	1	glucan_65_rpt	TIGR04035	5	endomucin	PF07010	1
F6-1 CDS_1158^c^	CGZ74_06870	glucan_65_rpt	TIGR04035	8						
F6-1 CDS_1205^c^	CGZ74_07120	glucan_65_rpt	TIGR04035	9						
F6-1 CDS_1215^c^	CGZ74_07170	Glyco_hydro_70	PF02324	1	glucan_65_rpt	TIGR04035	6			
F6-1 CDS_1217^c^	CGZ74_07180	glucan_65_rpt	TIGR04035	6						
F6-1 CDS_1218^c^	CGZ74_07185	Glyco_hydro_70	PF02324	1	glucan_65_rpt	TIGR04035	6	endomucin	PF07010	1
F6-1 CDS_1221^c^	CGZ74_07200	glucan_65_rpt	TIGR04035	5	RNase_E_G	PF10150	1			
F6-1 CDS_1222^c^	CGZ74_07205	glucan_65_rpt	TIGR04035	11						
F6-1 CDS_1223^c^	CGZ74_07210	glucan_65_rpt	TIGR04035	7	DUF4775	PF16001	1			
F6-1 CDS_1233^c^	CGZ74_07260	glucan_65_rpt	TIGR04035	4	DUF4775	PF16001	1			
F6-1 CDS_1249^c^	CGZ74_07340	Glyco_hydro_70	PF02324	1	glucan_65_rpt	TIGR04035	4			

^a^Classic signal peptide; ^b^signal peptide with YSIRK motif; ^c^signal peptide with KxKYxGKY. Domains indicated in bold are involved in adhesion or biofilm formation

### Identification of F6-1 LPxTG adhesins

**Fig. 3:**
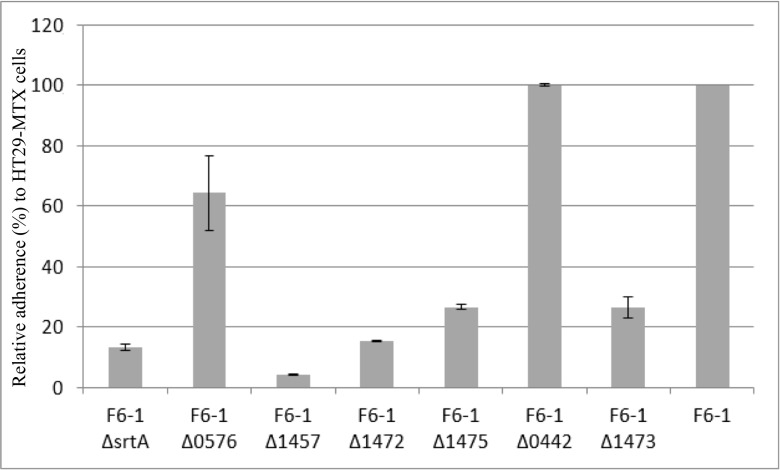
Adhesion capacity to HT29-MTX cell line of *S. salivarius* F6-1 and its seven mutants. The results are presented as a relative adherence calculated from the number of *S. salivarius* adhering bacteria (CFU per HT29-MTX cell) of each mutant compared to the wild type F6-1 which is standardized to 100%. Data represent means ± SEM from experiments performed with each strain in duplicate

In order to test the involvement of all the putative surface proteins containing an LPxTG motif in the adhesion to HT29MTX cells, the gene encoding the sortase A was replaced by an erythromycin resistance gene to generate the F6-1 *ΔsrtA* mutant. Its adhesion capacity to HT29-MTX cells (13.4 ± 0.9 CFU per HT29-MTX cell) is 7.5 times lower than that of F6-1 wild type (Fig. 3). This result suggests that at least some if not all the F6-1 surface proteins with an LPxTG motif are involved in the adhesion of F6-1 to HT29-MTX cells.

The 10 putative surface proteins with an LPxTG motif were analyzed to identify domains that are known to be involved in bacterial adhesion in other bacterial species (Table 2). Only the MucBP domain (PF06458) is known to be directly involved in bacterial adhesion to human intestinal epithelium. It is present as one copy in the F6-1 SALIVA_1472 protein of *S. salivarius* F6-1 (Table 2), whereas it is present as four copies in the MBF (mucus-binding factor) protein of *L. rhamnosus* GG, which is involved in its adhesion to the human intestinal mucus (von Ossowski et al. 2011). Seven other putative surface proteins contain domains which are not directly involved in adhesion to human intestinal epithelium but in adhesion to constituents of human body. For example, a collagen-binding domain (PF05737) is present as one copy in the F6-1 Big_3-4 protein of *S. salivarius* F6-1 (Table 2). It is known to be involved in the adhesion to collagen of *Staphylococcus aureus* strains (Symersky et al. 1997). The CshA-type fibril repeat (CshA_fibril_rpt, TIGR04225) is present as 27 copies in the F6-1 SALIVA_0893 protein of *S. salivarius* F6-1 (Table 2). It is involved in the adhesion to fibronectin of *S. gordonii* but only in the presence of another protein with a Hsa domain (Jakubovics et al. 2009). The serine-rich repeat adhesion glycoprotein AST domain (ser_adhes_Nterm, TIGR04224) is present as one copy in the F6-1 SALIVA_1456 and F6-1 SALIVA_1457 proteins of *S. salivarius* F6-1 (Table 2). It is known to be also involved in fibronectin linkage which allows adhesion to extracellular matrix and so to epithelium in streptococci (Handley et al. 2005). Other domains could be important for adhesion. For example, the glucan-binding protein C domain (GbpC; PF08363) is present as one copy in the F6-1 CDS_812 and F6-1 CDS_813 proteins of *S. salivarius* F6-1 (Table 2) and is known to be involved in adhesion to glucans (Sato et al. 1997). The G5 domain (PF07501) is present as two copies in the F6-1 SALIVA_1473 protein of *S. salivarius* F6-1 (Table 2). This domain is known to be involved in biofilm formation which involves adhesion as first step, as it has been demonstrated for the IgA1 peptidase of *Streptococcus pneumoniae* (Bateman et al. 2005). The sequence of the F6-1 SALIVA_1473 protein also shows 31% of identity with ZmpC from *S. pneumoniae* (data not shown), suggesting that it is a zinc metalloprotease. *Streptococcus* zinc metalloproteases are less described but Novak et al. (2000) showed their role in *S. pneumoniae* adhesion to the nasopharyngeal epithelium. Furthermore, ZmpC is known to be involved in *S. pneumoniae* adhesion to the eye mucins (Muc16) and to act as a virulence factor in this bacterium (Govindarajan et al. 2012; Menon and Govindarajan 2013).

Three F6-1 putative surface proteins with an LPxTG motif and domains that are known to be involved in bacterial adhesion were selected in order to test their involvement in F6-1 adhesion to HT29-MTX cells. The gene encoding the F6-1 SALIVA_1457 (ser_adhes_Nterm domain), F6-1 SALIVA_1472 (MucPB domain), and F6-1 SALIVA_1473 (G5 domains) proteins were separately replaced by a spectinomycin resistance gene to generate the F6-1 Δ1457, F6-1 Δ1472, and F6-1 Δ1473 mutants. As shown in Fig. 3, the adhesion capacities of these mutants are 23.0-, 6.6-, and 3.8-fold lower than that of F6-1, respectively. These significantly and statistically differences suggest that F6-1 SALIVA_1457, F6-1 SALIVA_1472, and F6-1 SALIVA_1473 are adhesins involved in the attachment of F6-1 to HT29-MTX cells.

### Identification of adhesins devoid of LPxTG motif

The 26 putative surface proteins devoid of LPxTG motif were analyzed to identify domains that could be involved in bacterial adhesion (Table 2). We have found three domains that are not involved in adhesion to human intestinal epithelium but that could be involved in adhesion to human extracellular matrix proteins. For example, the SSURE domain (PF11966) is present as three copies in the F6-1 CDS_925 of *S. salivarius* F6-1 and is found to bind to fibronectin but not to collagen or submaxillary mucin in *Streptococci* (Bumbaca et al. 2004). The FctA domain (PF12892) is detected as 4 and 17 copies in the F6-1 SALIVA_0576 and F6-1 SALIVA_1475 proteins of *S. salivarius* F6-1, respectively (Table 2). It is present in proteins involved in fibronectin and collagen binding of *Streptococcus pyogenes* and giving rise in some strains to pilus-like appendages involved in bacterial adherence and human host colonization (Manetti et al. 2007). The collagen-binding domain (PF05737) present as one copy in the F6-1 SALIVA_0576 protein of *S. salivarius* F6-1 (Table 2) is known to be involved in the adhesion to collagen of some *S. aureus* strains (Symersky et al. 1997). Other domains could participate to human colonization. For example, the Antigen_C domain (PF16364) is present as 10 copies in the F6-1 SALIVA_1475 protein of *S. salivarius* F6-1 (Table 2). It is present as two copies in SspB of *S. gordonii*, an antigen I/II family protein that is involved in host adhesion (Demuth et al. 1996). The GBS-Bsp-like domain (PF08481) is present in the F6-1 CDS_800, F6-1 SALIVA_0442, and F6-1 SALIVA_0518 proteins as 2, 2, and 4 copies, respectively (Table 2). It is present in Bsp of *Streptococcus agalactiae* that is involved in cellular morphology that impacts adhesion (Reinscheid et al. 2002). The SH3_5 domain (PF08460) is also present in the F6-1 SALIVA_0442 (6 copies) and F6-1 SALIVA_0518 (9 copies) proteins of *S. salivarius* F6-1 (Table 2). It is known to be involved in the interactions between proteins with proline-rich sequences, in cell-cell adhesion and in cell aggregation (Machiyama et al. 2015; Morton and Campbell 1994; Pawson and Schlessingert 1993).

Three of these F6-1 surface proteins were selected in order to test their involvement in the F6-1 adhesion to HT29-MTX cells. The genes encoding the F6-1 SALIVA_0442 (GBS_Bsp-like and SH3_5 domains), F6-1 SALIVA_0576 (FctA and collagen_bind domains), and F6-1 SALIVA_1475 (Antigen_C and FctA domains) proteins were separately replaced by a spectinomycin resistance gene to generate the F6-1 Δ0442, F6-1 Δ0576, and F6-1 Δ1475 mutants. As shown in Fig. 3, the adhesion capacities of the F6-1 Δ0442 and F6-1 Δ0576 mutants are not statistically different from that of F6-1wild-type (no difference for the F6-1 Δ0442 mutant; only a 1.6-fold decrease for the F6-1 Δ0576 mutant), suggesting that F6-1 SALIVA_0442 and F6-1 SALIVA_0576 are not involved in the F6-1 adhesion to the HT29-MTX cells. On the contrary, the adhesion capacity of the F6-1 Δ1475 mutant (26.7 ± 0.8 CFU per HT29-MTX cell) is 3.7-fold lower than that of F6-1 wild-type (Fig. 3), suggesting that F6-1 SALIVA_1475 is an adhesin involved in the attachment of F6-1 to HT29-MTX cells.

## Identification of homologs of putative adhesins in *Streptococci* genomes

Thus, our results show the involvement of four putative adhesins in the attachment of F6-1 to HT29-MTX cells, the three LPxTG proteins F6-1 SALIVA_1457, F6-1 SALIVA_1472, and F6-1 SALIVA_1473 and a protein devoid of LPxTG motif, F6-1 SALIVA_1475. Therefore, homologs of these F6-1 proteins were searched in all the translated CDSs of 33 *S. salivarius* sequenced genomes. These include 22 genomes already available in the NCBI database and 11 newly sequenced ones corresponding to *S. salivarius* strains tested in our aggregation/adhesion study.

The sequence of the F6-1 *SALIVA_1457* gene has two internal assembly gaps, which cannot be filled because of sequence repetitions. Such problem was also observed for 14 other sequences of F6-1 SALIVA_1457 homologs which are partial (data not shown). Thus, complete (7) or partial (14) sequences of F6-1 SALIVA_1457 homologs were detected in only 21 of the 33 strains (data not shown). The seven complete proteins have sizes between 1134 aa (HSISS4 strain) and 2300 aa (JIM8777 strain). They exhibit more than 86% of identity with F6-1 SALIVA_1457. They all include an LPxTG motif and a ser_adhes_Nterm domain (TIGR04224) as in F6-1 SALIVA_1457.

**Fig. 4:**
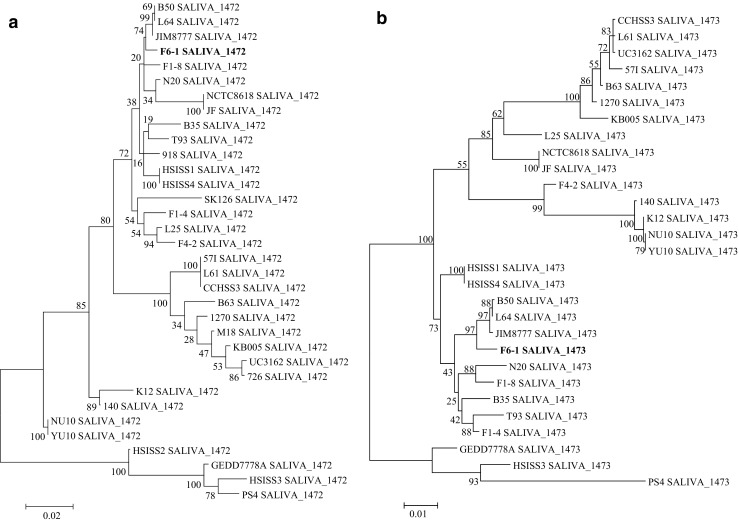
Phylogenetic ML tree obtained for putative adhesins of *S. salivarius* strains homologous to F6-1 SALIVA_1472 (**a**) and F6-1 SALIVA_1473 (**b**). The evolutionary history was inferred by using the maximum likelihood method based on the JTT matrix-based model. The tree with the highest log likelihood (− 9820.29) is shown. The percentage of trees in which the associated taxa clustered together is shown next to the branches. The tree is drawn to scale, with branch lengths measured in the number of substitutions per site. F6-1 SALIVA_1472 and F6-1 SALIVA_1473 are indicated in bold

Complete sequences of F6-1 SALIVA_1472 homologs were detected in all of the 33 other *S. salivarius* strains examined (Fig. 4a). These proteins have sizes between 1057 aa (strains KB005, 1270, and 726) and 1222 aa (strain HSISS4). They show more than 90% of identity with F6-1 SALIVA_1472. The most closely related proteins to F6-1 SALIVA_1472 are those of JIM8777, B50, and L64 strains (98.8% of identity). Almost all these proteins display an LPxTG motif as in F6-1 SALIVA_1472 except those of HSISS2, HSISS3, and PS4 strains. Furthermore, almost all these proteins show a MucBP domain as in F6-1 SALIVA_1472, except those of GED7778A, HSISS2, and HSISS3 strains.

Complete (28) or partial (5) sequences of F6-1 SALIVA_1473 homologs were detected in the 33 other strains examined (data not shown). The complete proteins have sizes between 1953 aa (strains 1270 and B63) and 2105 aa (strains F6-1, JF, and F1-4) and display more than 90% of identity with F6-1 SALIVA_1473. The proteins showing the closest relationship with F6-1 SALIVA_1473 are those of JIM8777, B50, and L64 strains (98.5% identity; Fig. 4b). All of these 28 proteins include an LPxTG motif and the same domains as in F6-1 SALIVA_1473 (one peptidase_M26_C, one peptidase_M26_N, and two G5).

The sequence of the F6-1 *SALIVA_1475* gene has two internal assembly gaps which cannot be filled because of sequence repetitions. Such problem was also observed for 16 other sequences of F6-1 SALIVA_1475 homologs which are partial (data not shown). In total, complete (17) or partial (16) sequences of F6-1 SALIVA_1475 homologs were detected in all the 33 *S. salivarius* strains (data not shown). The 17 complete proteins have sizes between 2220 aa (F1–4 strain) and 5423 aa (L25 strain). They show more than 70% of identity with F6-1 SALIVA_1475. All of these 17 proteins show the same domains as F6-1 SALIVA_1475 (Antigen_C and FctA), but their copy number is variable, from 2 (F1–4 strain) to 14 (B50 strain) for the Antigen_C domain and from 8 (F1–4 strain) to 20 (B50 strain) for the FctA domain.

## Discussion

Adhesion process of commensal bacterial strains to intestinal epithelial cells is an essential step in the colonization of human GIT. In this study, we demonstrated that the adhesion capacity to intestinal epithelial cell lines of *S. salivarius* strains varies considerably between strains. Such variability was previously observed with *S. thermophilus* on HT-29 (Couvigny et al. 2015) or *Lactobacillus* strains on Caco-2/TC7 cells (Jacobsen et al. 1999). Bacterial adhesion is known to rely on both nonspecific and specific ligands expressed at the bacterial surface that can interact with eukaryotic cell surface receptors. Therefore, our hypothesis is that differences in the charge, hydrophobicity, and/or structure of the surface of *S. salivarius* strain may account for their distinct adhesive properties.

The second finding is that most of the *S. salivarius* strains adhere better to HT-29 MTX cells than to Caco-2/TC7 cells as previously described for *L. rhamnosus* GG or other probiotic strains (Gopal et al. 2001). These two cell lines are characterized by distinct phenotypic traits. Caco-2/TC7 cells differentiate spontaneously in vitro forming a cell monolayer expressing apical and basolateral proteins as enterocytes of the small intestine but lacking mucus layer while HT29-MTX cells differentiate into goblet cells producing high amount of mucus mainly composed by MUC5AC mucins, predominantly produced in the stomach (Liévin-Le Moal and Servin 2013). Mucins are large glycoproteins with a protein backbone linked to a complex array of hydrophilic oligosaccharide side chains and represent potential ligands for microbial adhesions (Bansil and Turner 2006). Bacterial adhesion to the mucosal surface is considered as the first step for the successful colonization of the host surfaces. Previous studies demonstrated that the presence of mucus plays a major role in adhesion, likely due to the high adhesiveness of mucins present in the native human mucus layer covering the whole cell surface (Juge 2012; van Tassell and Miller 2011). Thus, the good adherence of some *S. salivarius* strains to HT29-MTX cells (for example strains F6-1 or F4-2) may reflect their excellent mucus-adhesive capacity which can be ascribable to strain-specific receptors at their surface.

Previous studies reported a relation between aggregation and adhesion ability for *Bifidobacterium longum* or *Lactobacillus reuteri* (Mackenzie et al. 2010; Del Re et al. 2000). However, our study of the adhesion/aggregation ability of 14 *S. salivarius* strains did not reveal such a correlation. Moreover, the deletion of the genes encoding the four putative adhesins significantly altered the adhesion ability of the mutants to HT29-MTX cells but had no impact on their aggregation properties (data not shown). Altogether, these results suggest that *S. salivarius* strains might perform their adhesion and aggregation by different mechanisms involving various surface molecules. This was observed for *Lactococcus lactis* BGKP1, in which the aggregation and the adhesion to gastric-type mucin proteins involve the aggregation factor AggL and the mucin-binding protein MbpL, respectively (Lukić et al. 2012, 2014).

Structure and composition of the cell wall determine surface properties which, in turn, influence adhesiveness. Previous studies reported that bacteria adapt to the GIT by different cell surface compositions or expression of genes encoding surface proteins, for example, in lactobacilli species (Golomb et al. 2016; Douillard et al. 2013; Azcarate-Peril et al. 2008; Denou et al. 2007; Pridmore et al. 2004). Accordingly, the absence of sortase in *Lactobacillus salivarius* is associated with a reduced capacity of adhesion to Caco-2/TC7 cells (Jensen et al. 2014). In this study, we showed that the removal of sortase significantly reduced the *S. salivarius* F6-1 adhesion to HT29-MTX cells, and therefore, we demonstrated that sortase-dependent proteins play an active role in host adhesion. Previous analyses indicated that *S. salivarius* is rich in genes encoding surface factors (Delorme et al. 2015). Our analysis of the genome of *S. salivarius* F6-1 is in agreement with this observation since 36 genes encoding putative subcellular proteins were detected. This high proportion of genes encoding surface factors suggests that extracellular components confer *S. salivarius* an adequate fitness to the GIT either by increasing their residence time in the gut, or by facilitating their interactions with host components or with other resident bacteria. Indeed, a few *S. salivarius* extracellular components have already been demonstrated to promote interaction with host (Schenkels et al. 1993; Handley et al. 1987) and/or with endogenous microbiota (Lévesque et al. 2003; Handley et al. 1987; Weerkamp and Jacobs 1982).

**Fig. 5:**
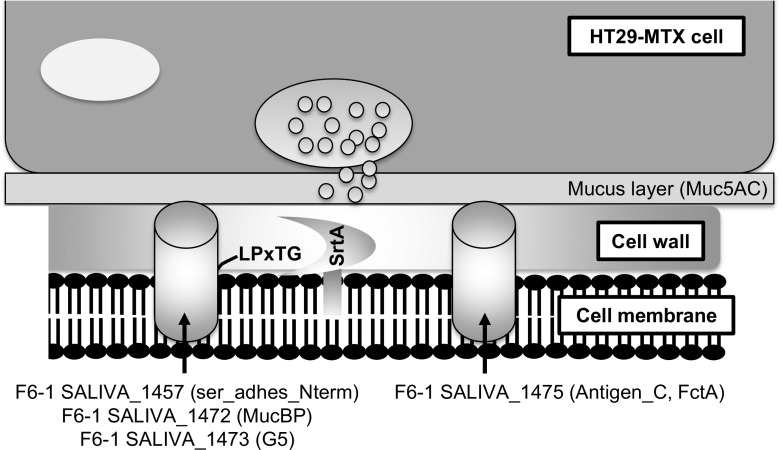
Involvement of SrtA and adhesins in the adhesion of *S. salivarius* F6-1 to HT29-MTX cell line. The cylinders correspond to F6-1 adhesins identified in this work. The name of the proteins and of their potential adhesive domains are indicated

In this work, four *S. salivarius* F6-1 genes were demonstrated to encode putative adhesins, their deletion being associated with a significant decrease in adhesion ability to HT29-MTX cells of the mutants compared to WT strain (Fig. 5). One is the F6-1 *SALIVA_1472* gene that encodes a protein with a MucBP domain which may interact with intestinal mucus (Boekhorst et al. 2006). Homologs of this adhesin are encoded by all the *S. salivarius* genomes analyzed. Numerous proteins containing MucBP domains have been found in lactic acid bacteria and predominantly in lactobacilli found naturally in intestinal niches (van Tassell and Miller 2011). MucBP domains are also present as four copies in a protein of *L. lactis* subsp*. cremoris* IBB477 involved in adhesion to bare and mucin-coated polystyrene (Radziwill-Bienkowska et al. 2016) or as three copies in MbpL of *L. lacti*s BGKP1 that promotes adhesion to HT29-MTX cells (presumably to the Muc5AC) and pig gastric mucins (Lukić et al. 2012).

The F6-1 *SALIVA_1457* gene of *S. salivarius* F6-1 encodes a protein with a serine-rich repeat adhesion glycoprotein AST domain. Homologs of this adhesin are encoded by only 21 of the 33 *S. salivarius* genomes analyzed. Such domain has been found in serine-rich repeat glycoproteins (SRRPs), a family of glycosylated adhesins highly conserved in Gram-positive bacteria and mediating bacterial-host interactions (Zhu and Wu 2016). For example, the surface protein Srr-1 of *S. agalactiae* binds human keratin 4 of saliva and fibrinogen and promotes adherence to epithelial HEp-2 cells of larynx (Samen et al. 2007; Seo et al. 2013). In *Streptococcus parasanguinis*, the Fap1 protein is the major subunit of the long fimbriae and is crucial for biofilm formation (Froeliger and Fives-Taylor 2001). In *S. gordonii*, the GspB and Hsa proteins bind to salivary glycoproteins such as MG2, salivary agglutinin, and secretory IgA (Takamatsu et al. 2006).

The F6-1 *SALIVA_1473* gene of *S. salivarius* F6-1 encodes a protein with two G5 domains. Homologs of this adhesin are encoded by all the *S. salivarius* genomes analyzed. The implication of proteins with such domains in adhesion to human intestinal epithelial cells has not yet been reported. However, proteins with such domains are required for biofilm formation, for example, in *Staphylococcus epidermidis* and *S. aureus* (Aap and SasG proteins, respectively). Furthermore, biofilm formation is dependent on the copy number of G5 domains; five or more G5 domains are required in SasG to support biofilm formation of *S. aureus* (Corrigan et al. 2007).

The F6-1 *SALIVA_1475* gene encodes a protein with 10 Antigen_C and 17 FctA domains. Homologs of this adhesin are encoded by all the *S. salivarius* genomes analyzed. Such Antigen_C domain has been found in polypeptides of the antigen (Ag) I/II family that are cell wall-anchored proteins produced by most indigenous species of oral streptococci (Brady et al. 2010). These adhesins have been shown to bind a wide range of host cell proteins such as collagen, fibronectin, or fibrinogen (Beg et al. 2002) and salivary agglutinin gp-340 (Jakubovics et al. 2005). In addition, the Ag I/II family polypeptide SspB from *S. gordonii* has been shown to interact directly with other microorganisms such as *Actinomyces naeslundii* (Jakubovics et al. 2005). Moreover, Sal_2056 and BspA of *S. agalactiae* contain two copies of this Antigen_C domain and are, respectively, involved in biofilm formation (Chuzeville et al. 2015) and in adhesion to scavenger receptor gp340, human vaginal epithelium, and to the fungus *Candida albicans* (Rego et al. 2016).

In this work, we showed the high diversity of the putative surface proteins of *S. salivarius* F6-1, a strain that strongly adheres to human intestinal epithelial HT29-MTX cells. Such diversity has already been described in *L. lactis* subsp. *cremoris* IBB477 (Radziwill-Bienkowska et al. 2016), but only two common protein domains (MucBP and SSURE) have been detected. Among these F6-1 putative surface proteins, we have identified four adhesins, which are also present, for three of them (F6-1 SALIVA_1472, F6-1 SALIVA_1473, and F6-1 SALIVA_1475), in all other *S. salivarius* strains analyzed. This work increases knowledge on adhesins of *S. salivarius* but it is not exhaustive. Bacterial interaction with host cells is a complex and dynamic process involving a variety of bacterial cell surface structures and host receptors. In addition to these specific bacterial adhesins, other cell surface molecules such as S-layer proteins, lipoteichoic acid, or exopolysaccharides (Lebeer et al. 2008) and extracellular appendages such as fimbriae, fibrils, or pili (Juge 2012) can also contribute to adhesion to host epithelial cells and mucus. Further work is needed to evaluate their contribution to *S. salivarius* adhesion.

## Electronic supplementary material

ESM 1 (PDF 936 kb)
