# Supplementary material for: Surface proteins involved in the adhesion of Streptococcus salivarius to human intestinal epithelial cells
Source: Appl Microbiol Biotechnol. 2018 Feb 13;102(6):2851–65. doi: 10.1007/s00253-018-8794-y (PMC5847202; doi:10.1007/s00253-018-8794-y)
Supplement: Supplementary file 1 — (PDF 936 kb) [file 253_2018_8794_MOESM1_ESM.pdf]

**Supplementary materials**

Applied Microbiology and Biotechnology

**Surface proteins involved in the adhesion of *Streptococcus salivarius* to human intestinal epithelial cells**

Fanny Chaffanel[1], Florence Charron-Bourgoin[1], Claire Soligot[2], Mounira Kebouchi[2], Stéphane Bertin[1], Sophie Payot[1], Yves Le Roux[2], Nathalie Leblond-Bourget[1 #]

[1] DynAMic, Université de Lorraine, INRA, 54506, Vandoeuvre-lès-Nancy, France

[2] UR AFPA, équipe PB2P, Université de Lorraine, INRA, 54506, Vandoeuvre-lès-Nancy, France

# Corresponding author

Tel: +33 3 72 74 51 46

Fax: +33 3 72 74 53 56

E-mail address: nathalie.leblond@univ-lorraine.fr

**Table S1: Primers used for the construction of *S. salivarius* F6-1 mutants.**

[a] primers used for amplication of the spectinomycin resistance gene of the pSL1180 spec lox plasmid

[b] primers used for amplication of the *Spe*I-*Spe*I spectinomycin resistance cassette from the pSET4s plasmid

**Fig. S1: Strategy of *S. salivarius* F6-1 mutants construction by allelic replacement.**

The spectinomycin (*spec*) or erythromycin (*ery*) resistance genes were amplified from plasmids by PCR using specific primers represented by black arrows. In parallel, two DNA fragments overlapping the upstream (1) and downstream (2) regions of the chromosomal target gene were amplified by PCR using specific primers that present an extended sequence matching with the 3' and 5' ends of the resistance gene. A second PCR amplification was carried out using these two PCR fragments and the resistance gene as template to synthesize an overlap PCR product carrying *spec* or *ery* flanked by the chromosomal regions 1 and 2. This final PCR product was used to transform the *S. salivarius* F6-1 strain. The crossover events represented by crosses, upstream and downstream from the target gene, were positively selected by the newly acquired antibiotic resistance (Spec$^R$ or Ery$^R$) of the transformed clones.

**Table S1: Primers used for the construction of *S. salivarius* F6-1 mutants**

| Primers | Nucleotide sequences (5'-3') | Product size (bp) |
|---|---|---|
| **F6-1 sortase_1** | GCATGTTCGATACCAAGTG | 975 |
| **F6-1 sortase_2** | TAAGAAAGGGGCCCTCGG | |
| **F6-1 sortase_3** | TAGTTGTCACCACTTTCTTGA | 1027 |
| **F6-1 sortase_4** | GCACAGAGCTTATGGTCG | |
| **gyr-eryF** | TCAAGAAAGTGGTGACAACTAATGAACAAAAATATAAAATATTCTCA | 774 |
| **eryRlyase** | CCGAGGGCCCCTTTCTTATTTCCTCCCGTTAAATAATAG | |
| **F6-1_SALIVA_0442.1** | ACAGCTGGAGTAAATCAAGG | 1000 |
| **F6-1_SALIVA_0442.2** | TTGGGAAATATTCATTCTAATTGGGGCTGTTTGATAAAAATTGG | |
| **F6-1_SALIVA_0442.3** | CCAGTCACGTTACGATAACTTCGCGACGTTATGTAGCTGTG | 1016 |
| **F6-1_SALIVA_0442.4** | TCTACATAGCGTCTTGCAC | |
| **SALIVA_0576.1** | TTCGGACTTAAGGTTACCA | 1038 |
| **SALIVA_0576.2** | TATTTAATATTTGGGAAATATTCATTCTAATTGGGGTTTCCCCTAAGTCAACAG | |
| **SALIVA_0576.3** | AATTTAGTTTATTTATAGATTTCATTGGCTTCTAGCTGCTGTTTCTATCACGGT | 1038 |
| **SALIVA_0576.4** | GCGACGATGGCTATATGCT | |
| **SALIVA_1457.1** | GAATCCGTCTGACTTACTGAT | 996 |
| **SALIVA_1457.2** | TATTTAATATTTGGGAAATATTCATTCTAATTGGCCAAGCGCTTCCTAAGACT | |
| **SALIVA_1457.3** | AATTTAGTTTATTTATAGATTTCATTGGCTTCTACCCGTCATTTGTCACTTTCA | 1020 |
| **SALIVA_1457.4** | GAAACTGATCGCGTGACTCG | |
| **SALIVA_1472.1** | CTGTTACTGGCAATTGTTCAT | 1017 |
| **SALIVA_1472.2** | TATTTAATATTTGGGAAATATTCATTCTAATTGGAACCAGGAACACCAATCTA | |
| **SALIVA_1472.3** | AATTTAGTTTATTTATAGATTTCATTGGCTTCTACCACTTGTTTCATCACGGTA | 1274 |
| **SALIVA_1472.4** | GAACTTCCACCAGTACCAGC | |
| **F6-1 SALIVA_1473.1** | CTTCGGCTACTGCTGTTGAG | 996 |
| **F6-1 SALIVA_1473.2** | CCAGTCACGTTACGATAACTTCGGGTGTCAACAAGCCATACG | |
| **F6-1 SALIVA_1473.3** | TTGGGAAATATTCATTCTAATTGGGGAGCACAGCCATAGTCG | 1013 |
| **F6-1 SALIVA_1473.4** | GAGGAGATTTCAGGGAGCG | |
| **SALIVA_1475.1** | TCAGCTGAATCGTCGTCATC | 1071 |
| **SALIVA_1475.2** | TATTTAATATTTGGGAAATATTCATTCTAATTGGCGTTCTTAGCGATGGTAACG | |
| **SALIVA_1475.3** | AATTTAGTTTATTTATAGATTTCATTGGCTTCTACAACGATTGCTTTAGCTGT | 1051 |
| **SALIVA_1475.4** | ACAGTTGAAGTTGCTAAGAA | |
| **Spec Fwd**[a] | TAGAAGCCAATGAAATCTAT | 936 |
| **Spec Fwd_bis**[a] | CGAAGTTATCGTAACGTGAC | 1083 |
| **SpecRev**[a] | CCAATTAGAATGAATATTTCCC | |
| **Spec-lox71-SpeI F**[b] | TTTTTACTAGTTCGTACCGTTCGTATAGCATACATTATACGAAGTTATCGTAACGTGACTGGCAAGA | 1169 |
| **Spec-lox66-SpeI R**[b] | TTTTTACTAGTCGTACCGTTCGTATAATGTATGCTATACGAAGTTATCCAATTAGAATGAATATTTCCC | |

[a] primers used for amplication of the spectinomycin resistance gene of the pSL1180 spec lox plasmid

[b] primers used for amplication of the *Spe*I-*Spe*I spectinomycin resistance cassette from the pSET4s plasmid

**Fig. S1**

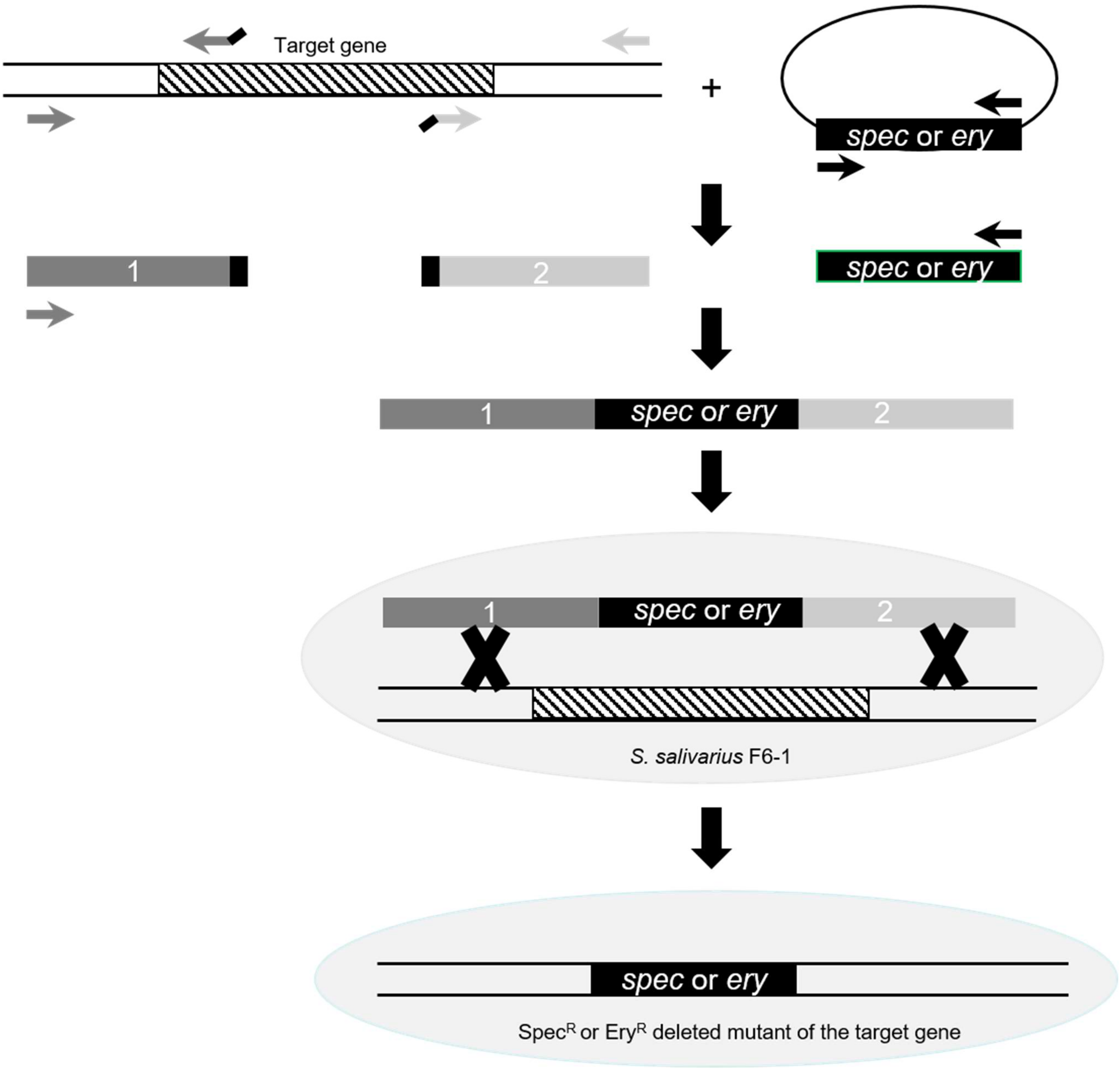

Target gene
+
spec or ery
1
2
spec or ery
1
spec or ery
2
1
spec or ery
2
S. salivarius F6-1
spec or ery
Spec^R or Ery^R deleted mutant of the target gene
